# The Disadvantaged Home Care Personal Support Worker: Differences in Job Characteristics, Unionization, Pensions, Participation, and Wages by Care Sector in Canada

**DOI:** 10.1177/07334648221146260

**Published:** 2022-12-16

**Authors:** Katherine Zagrodney, Raisa Deber, Mike Saks, Audrey Laporte

**Affiliations:** 1 Institute of Health Policy, Management and Evaluation, 7938 University of Toronto, Toronto, ON, Canada; 2 Canadian Centre for Health Economics (CCHE), Toronto, ON, Canada; 3 612784 VHA Home HealthCare, Toronto, ON, Canada; 4 Canadian Health Workforce Network (CHWN), Ottawa, ON, Canada; 5 6363 University of Ottawa, Ottawa, ON, Canada; 6 102099 University of Suffolk, Ipswich, UK; 7 4547 University of Lincoln, Lincoln, UK; 8 4921 University of Westminster, London, UK

**Keywords:** employment, health services, healthcare workforce, home care, home and community based care and services, long-term services and supports, personal support workers, personal support worker wages

## Abstract

Personal Support Worker (PSW) supply is struggling to match the rising demand within many countries, particularly in the home and community (HC) sector. Although care demand projections are often sector-specific, our understanding of sector discrepancies on the PSW labour supply side is limited. This paper compares PSW job characteristics by means, proportions, and tests of significance across HC, nursing and long-term care home (LTC), and hospital sectors utilizing a sample of Canadian PSWs (1996–2010). Compared to LTC and hospital sectors, HC PSWs had significantly lower average wages, labour participation levels, permanent positions, job duration, and unionization rates. Relative wage distribution graphs showed how sector wage discrepancies existed across the wage distribution. These distinctions made the comparatively disadvantaged HC PSW position particularly salient, with important labour supply implications by sector. The relative attractiveness of HC sector jobs will become more critical as the rise in HC demand is projected to continue.

**What this paper adds**

• Direct comparisons of PSW job characteristics across hospital versus LTC versus HC sectors were provided in a single study with a large, national, longitudinal sample of PSWs.
• Several methods were used to provide evidence of wage differences by sector; with HC PSWs receiving the lowest wages, followed by LTC PSWs, and hospital PSWs the highest.
• Multiple job-related factors significantly differed by sector, where the generally more desirable conditions (e.g., permanent employment, unionization, pension plans) were less common in HC compared to other sectors.

**Applications of study findings**

• Significant differences in labour characteristics by sector suggest that policies would be most effective if they treated each sector as inter-related sub-markets, rather than an all-encompassing homogeneous PSW labour market.
• Advocates for the HC sector can utilize these findings as evidence that HC PSWs have received lower wages than PSWs in LTC or hospital sectors.
• Beyond wages, improvements to PSW positions such as increasing full-time employment, full-year employment, and employer-based pension plans are particularly relevant to HC PSWs.

## Introduction

Personal Support Workers (PSWs) provide frontline care tasks across home and community (HC), nursing and long-term care homes (LTC), and hospital sectors. There are many alternative titles for PSWs (e.g., Health Care Aides (HCAs) and Direct Care Workers (DCWs)), where use of a given title often varies by region, sector, and/or employer. Personal Support Workers also have a wide scope of practice, often providing assistance with activities of daily living (e.g., bathing), amongst tasks that are both more and less medically aligned. Personal Support Workers typically work as part of a team of healthcare professionals, often including nurses; yet, PSWs differ from many other frontline healthcare workers in that they are not a recognized regulated health profession even though they are essential to patient care. The precarious nature of the PSW workforce is demonstrated through common features such as low wages, limited job mobility, and contract-based employment (Zagrodney & Saks, 2017).

Heightened PSW utilization has occurred over time and is likely to persist, as forecasts estimate a substantial and continual increase in demand for PSWs into the future (Toews, 2016). Contributing factors to increased PSW utilization likely include the growing patient population requiring help with activities of daily living, such as those with co-morbidities and an older population (Health Council of Canada, 2012). This shift in patient needs towards the type of services that PSWs provide is paired with pressures to keep the cost of care provision low; both contribute to the increased PSW utilization—as PSWs tend to receive lower wages than other healthcare workers, such as nurses. But, as the role of PSWs in the delivery of care expanded, it has been a challenge for PSW supply to match demand accordingly. Personal Support Worker shortages, retention, and recruitment were already major health human resource challenges across multiple countries prior to the COVID-19 pandemic (Fujisawa & Colombo, 2009), which will likely have further implications for PSW supply.

Projections for PSW demand are often specific to hospital, LTC, or HC care sectors (Home Care Sector Study Corporation, 2003; Toews, 2016). Furthermore, healthcare planning and funding is often separated by care sector, as is the case in Canada. This contributes to differences in funding processes and distributions by sector, with possible implications for job-related factors such as wage rates even for publicly funded PSWs. Gaining a better understanding of the potential job-related differences by sector will help to tailor future PSW labour supply policy planning for each sector. Although many workers across HC, LTC, and hospital sectors fall under the PSW umbrella term—often sharing job titles, educational training programs, and performing similar tasks at work—job-related factors may differ by employer and/or sector. It follows that PSW supply information specific to each sector is of high priority for health workforce policy to ensure adequate PSW supply not just as a whole, but to match demand for PSW services specific to each sector.

Literature addressing PSW labour supply by care sector is relatively limited. Indeed, comparisons across sectors are scarce and what is available is largely from the US (e.g., Yamada, 2002) or drawn from comparisons across different sector-specific samples (e.g., Zeytinoglu, et al., 2009). Comparing across sector-specific findings from available Canadian literature, we anticipate sector differences in PSW job characteristics and wages, and hours worked. Past research suggests potential sectoral differences in job characteristics such as full-time (FT) employment status (Montgomery et al., 2005), permanent versus temporary job positions (Pyper, 2004), job tenure (Hewko et al., 2015), unionization (Dill et al., 2012), and benefits and pensions (Home Care Sector Study Corporation, 2003). Hours worked can also be expected to vary by sector (Dill et al., 2012; Home Care Sector Study Corporation, 2003; Montgomery et al., 2005). Wages are of particular importance to PSW supply; low PSW wages have been cited as a major influencing factor for exit from the PSW occupation entirely and/or of PSWs from the HC sector (Zeytinoglu et al., 2009). Based on available literature, hospital PSWs are expected to generally be paid the highest and HC PSWs the lowest (Lilly, 2008; Montgomery et al., 2005).

This paper adds to the existing literature by providing a detailed comprehensive comparison of PSWs in the hospital versus LTC versus HC sectors in terms of job characteristics, hours worked, and wages on a pan-Canadian level. More specifically, the first aim was to utilize a longitudinal province-wide Canadian dataset that has not previously been employed in the known literature to provide descriptive statistics and tests of significance by sector for PSW participation (number of paid hours, employment status, job schedule, permanent status), employment history (job duration, jobless spells, number of weeks worked, year-round work), unionization, employer characteristics (size of employer, self-employment), pensions, and wages. Given the importance of wages for this workforce, our second research aim was to utilize the relative distribution approach developed by Handcock and Morris (2006) to provide a more detailed wage comparison beyond means and reports of central tendency. This method allows for a detailed comparison of wages by sector that has not previously been conducted for PSWs. Instead of relying on averages alone, these graphs provide more insight into which sector was more likely to have received wages within a specific wage range. Details obtained from these relative wage distribution graphs will provide important information for policy. For example, we could see whether it was the case that although LTC PSW wages were significantly lower than hospital wages on average, that LTC PSWs were more likely to receive wages in the mid-range. Overall, knowledge derived from this research aims to improve our understanding of the PSW labour market by providing descriptive statistics separated by care sector.

## Methodology

### Data

The data utilized was a sample of PSWs from the Survey of Labour and Income Dynamics (SLID). The SLID dataset was based on a sample of the Canadian population matching proportions of the total country by province, but excluded areas such as Nunavut, Indian reserves, etc. due to privacy issues of relatively small populations (Statistics Canada, 2013b). Occupational codes for primary employment were used to identify survey respondents working as PSWs (see the Working Sample section below). The SLID utilized a longitudinal panel structure, which captures annual information on groups of individuals (panels consisting of approximately 34,000 participants) for six consecutive years per panel from 1996–2010 (the last year in which a full panel was conducted). Therefore, one person can represent up to six PSW years. Despite the less recent time-period captured in the dataset (1996–2010), the SLID was utilized in absence of any alternative health workforce data sources for PSWs in Canada with this level of detail and ability to compare across sectors. Moreover, the set of factors associated with differences in patterns of supply are expected to apply across time. Following Statistics Canada recommendations, longitudinal weighted sample sizes were used for all results to ensure population representativeness and participant anonymity. Utilizing weighted survey data assumes that results from survey sampling of part of the population can be extrapolated and generalized to represent the entire population. Weights are generated by Statistics Canada and applied to survey participants so that a single individual in the survey can more accurately reflect the multiple people from the larger population who they represent (Statistics Canada, 2013b).

### Working Sample

The sample of PSWs utilized in this paper were identified by the following National Occupation Classification for Statistics (NOC-S) codes for primary employment in that year: G811 for “Visiting Homemakers, Housekeepers and related occupations” (e.g., home health aide, PSW) and D312 for “Nurse aides, Orderlies and Patient Service Associates” (e.g., health care aide, long-term care aide) (Statistics Canada, 2016a, 2016b, 2016c). Table 1 outlines how NOC-S and industry codes were used to classify PSWs into sectors; with LTC and hospital falling under D312 and further split by industry code and HC under G811 with industry codes listed in Table 1.

**Table 1.** Statistics Canada Industry Code and Description by Sector.

Nursing Homes and Long-Term Care Homes (LTC) (NOC-S D312)
6230: Nursing and residential care facilities n.e.c.
Hospital (NOC-S D312)
6219: Other ambulatory health care services
6220: Hospitals (all types of care)
Home and community (HC) (NOC-S G811)
6241: Individual and family services
6216: Home health care services
8141: Private households
8131: Religious organizations
6214: Out-patient care centres
6243: Vocational rehabilitation services
6213: Offices of other health practitioners
5617: Services to buildings and dwellings
6242: Community food and housing, and emergency and other relief services
8132: Grant-making and giving services
6211: Offices of physicians
8133: Social advocacy organizations
8134: Civic and social organizations
6244: Child day-care services
4461: Health and personal care stores

Survey respondents could enter/exit the PSW workforce or a given sector annually and could represent up to six PSW years, dependent on their primary employment in a given year. For example, if an individual’s primary employment was as a HC PSW in year one, unemployment in year two, and LTC PSW in year three, the individual would be counted as a HC PSW in the first year, not included in the sample in the second year, and part of the LTC PSW sample in their third year. Primary employment was used because the SLID only captured detailed job-related characteristics corresponding to the primary job. This working sample is referred to as PSWs throughout the paper, but we recognize that survey respondents under these NOC and Industry codes can provide a broad range of care tasks which were not captured in the SLID.

### Descriptive Statistical Methods

Means and proportions for each of the outlined labour supply and job characteristics were reported. In addition, one-way ANOVA was used to test for statistically significant differences for each variable by sector (hospital vs. LTC vs. HC).

### Relative Distribution Methods

The relative distribution approach provides percentiles of given wage values that one group (the comparison group) would have if placed within the distribution of the reference group (Handcock & Morris, 2006); in this case, we were comparing wage distributions of PSWs between any two sectors. This technique allowed for examination of wages across the entire distribution non-parametrically, where discrete changes at various thresholds were determined. This is an important distinction from typical reports of the mean, as details concerning where and how wages differ between two groups are hidden when utilizing averages alone.

The following explanation of the relative distribution approach is presented in line with Handcock and Morris (2006). Here, the same random variable is observed in both the reference group, $Y_{0}$ and the comparison group, *Y*, respectively. Then, following Handcock and Morris (2006), let the relative distribution of the random variable, *R*, for both groups be defined by

(1) $$R=F_{0}\left(Y\right)$$

Thus, *R* is a representation of the percentile position or quantile of the comparison group where a value of the comparison group lies if it were placed within the reference group’s distribution. As a random variable, *R* has both a probability density function (PDF) and a cumulative distribution function (CDF). The CDF for *R* (alternately defined as the quantile function of $F_{0}$) is given as:

(2) $$G\left(r\right)=F\left(F_{0}^{-1}\left(r\right)\right),0\leq r\leq 1$$

Where r is any point on the CDF of the reference group $Y_{0}$. The PDF for *R* is then $G{\left(r\right)}^{\prime }$ s derivative with respect to r, shown as:

(3) $$g\left(r\right)=\frac{f\left(F_{0}^{-1}\left(r\right)\right)}{f_{0}\left(F_{0}^{-1}\left(r\right)\right)},0\leq r\leq 1$$

Equation (3) is the basis for the relative distribution approach as it shows the ratio of densities between groups in reference to the quantiles of the reference group. It is both a density ratio as well as a PDF as it integrates to 1 by rescaling the quantile function. The values of g(r) are equal to 1 when no differences in distributions between groups are present.

When applying this relative distribution approach, the results are displayed through graphs which distinguish the points at which the reference and the comparison group differ along the wage distribution. The display of the PDF in a single graphical representation allows for ease of interpretation and shows at which points wage values were more likely to be in either the comparison (values less than 1) or the reference (values greater than 1) groups. Such distinctions are potentially important for policy and planning purposes and are not available through reports of averages alone. Relative distribution graphs were generated using R software (R Core Team, 2019).

## Results

### Sample Description

The total sample consisted of approximately 6,000 PSW years over the entire sample period without accounting for weights, which equates to approximately 2,179,400 datapoints once longitudinal survey weights were applied. The majority of PSWs in this sample worked in the LTC sector (46.20%, *n* = 1,006,800 weighted, or approximately 2700 PSW years unweighted), followed by HC (32.66%, *n* = 711,700 weighted, or approximately 2000 PSW years unweighted), and the hospital (21.15%, *n* = 460,900 weighted, or approximately 1200 PSW years unweighted). These proportions by sector are comparable with prior reports (Government of Ontario, 2011), suggesting that the sample appropriately reflects sector distribution.

There were more females employed as LTC (88.5%) or HC (84.7%) PSWs compared to hospital PSWs (66.3%). Average ages across sectors ranged from 41 in LTC, to 42 in hospital, and 43 in HC. In this sample, immigrant status was highest for hospital PSWs (23.9%), followed by HC PSWs (23.4%) and LTC PSWs (17.6%). Hospital PSWs had the highest proportion of education above high school (13.3%) versus HC (9.30%) or hospital (9.20%). For further information about the sample, see Zagrodney et al. (2022).

### Labour Market Participation and Hours Worked Characteristics

Personal Support Workers in our sample worked an average of 1455.8 paid hours per year (*SD* = 724.6); at the higher end of ranges reported for PSWs internationally and with more recent data (611–1,786 hours (Hewko et al., 2015)). Hospital PSWs worked the most paid hours per year on average (*M* = 1,546.4, *SD* = 573.4) versus LTC (*M* = 1,503.8, *SD* = 732.7) or HC PSWs (*M* = 1,329.3, *SD* = 782.0); the differences across sectors were significant (*F* (2, 2,179,397) = 46.65, *p* < .01), contrasting with previous US findings reporting small sectoral differences (Crown, et al., 1995; Montgomery et al., 2005).

**Figure 1.**
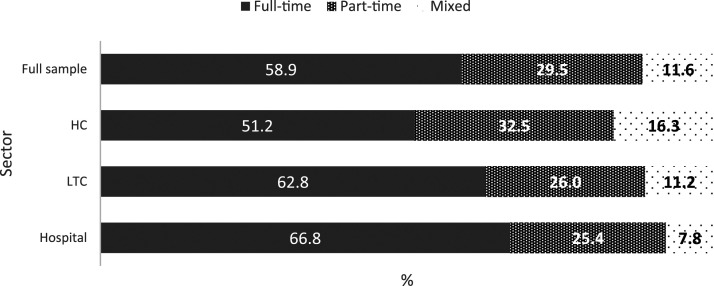
Distributions of full-time versus part-time employment status’ by sector.

Although the majority of PSWs worked exclusively FT (58.90%), over one-third worked part-time (PT) at some point in the reference year (41.10%). A part-time job is defined in the SLID as working less than 130 hours per month (Statistics Canada, 2013a). There were significant differences in FT versus PT work by sector (*F* (2, 2,179,397) = 52.44, *p* < .01). Hospital PSWs had the lowest proportions working “PT” or “some FT, some PT” (33.2%) versus LTC (37.2%) or HC (48.8%) (proportions derived from the sum of values “PT” and “some FT, some PT” in Figure 1 below). The proportion of PT HC PSWs matches well with findings in the literature (e.g., 37% in prior literature (Zeytinoglu et al., 2009) vs. 32.5% in this sample).

Most PSWs were employed full-year (82.80%) while almost one-fifth (17.23%) were employed part-year. A full-year can include FT or PT and is defined by Statistics Canada as employment for 53 weeks in a year. Hospital PSWs had the highest proportions of full-year employment (89.1%) versus LTC (84.8%) or HC (74.5%); these sectoral differences were significant (*F* (2, 2,179,397) = 67.23, *p* < .01). There were higher proportions of HC PSWs with more than one job (27.7%) versus LTC (19.3%) or hospital (15.5%) PSWs.

There were significant sectoral differences in permanent positions (*F* (2, 2,179,397) = 9.2, *p* < .01). The proportion of LTC and hospital PSWs with permanent positions were comparable (86.4% and 85.5%, respectively), while permanency rates were lower for HC PSWs (74.0%).

Although most PSWs were satisfied with the number of weeks worked in the reference year or wanted to work less (80.5%), almost one-fifth wanted to work more (19.5%). The proportions of PSWs wanting to work more weeks significantly differed by sector (*F* (2, 2,179,397) = 5.7, *p* < .01); where comparably more PSWs in LTC (21.6%) and HC (21.1%) wanted to work more weeks than did hospital PSWs (9.1%).

**Figure 2.**
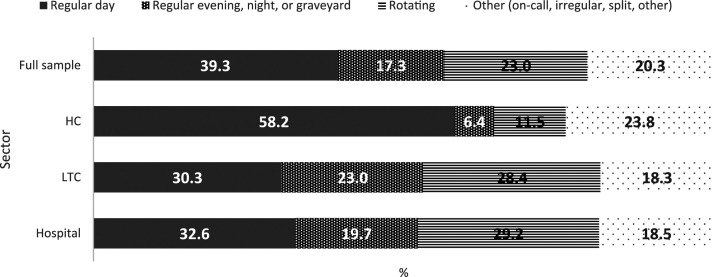
Distributions of shift schedule types by sector.

That the majority of PSWs in our sample worked a day shift (39.3%) and that regular evening, night, or graveyard shifts were less common (17.3%) mirrors previous literature (Hewko et al., 2015). Approximately one-fifth (20.1%) of PSWs worked an irregular schedule (on-call, irregular, split, other), while approximately one-quarter (23.3%) had a rotating shift schedule. Shift schedules significantly differed by sector (*F* (2, 2,179,397) = 26.0, *p* < .01); distributions were fairly even across shift types for hospital and LTC PSWs, but HC PSWs had higher proportions working a regular day shift or “other” shift (Figure 2).

### Employment History

Average tenure with the same employer (job tenure) across the sample was 9.14 years; the highest rate reported in an international scoping review (ranging from 2.16–9.86 (Hewko et al., 2015)). Proportionally, 40.4% of PSWs had worked for their employer for 5 years or less. Average job tenure in months was lowest for HC PSWs (*M* = 82.4, *SD* = 75.7) versus LTC (*M* = 107.8, *SD* = 92.8) or hospital PSWs (*M* = 156.4, *SD* = 122.3), with significant differences by sector (*F* (2, 2,179,397) = 263, *p* < .01) suggesting higher HC turnover.

Approximately one-fifth (17.1%) of PSWs had a jobless spell at some point within the same year that they worked as a PSW. There were significant differences by sector (*F* (2, 2,179,397) = 74.6, *p* < 0.01): approximately one-quarter of HC PSWs (24.9%) reported one or more jobless spells in a year—comparably higher than LTC (14.9%) or hospital (10.1%) PSWs.

### Unionization

The majority of PSWs were unionized (62.8%), which corresponds well with other Canadian PSW samples (Dill et al., 2012). The majority of hospital (93.4%) and LTC (68.4%) PSWs were unionized compared to only one-third of HC PSWs (35.0%); these sector differences were significant (*F* (2, 2,179,397) = 818.0, *p* < .01).

### Pensions

Approximately half (52.5%) of PSWs received a pension from their employer. Hospital PSWs were more likely to have a pension plan (82.9%) versus LTC (55.3%) or HC (28.8%) PSWs and sectoral differences were significant (*F* (2, 2,179,397) = 576.5, *p* < .01) (consistent with previous US literature (Yamada, 2002)).

### Wage and Income

The average hourly wage for this sample was $14.0 (CAD) (SD = 5.2). All wages and income were adjusted for inflation to the year 2006; given the longitudinal SLID data (1996–2010), adjusting for inflation towards a mid to upper limit point more accurately reflects the wages across the time-period. This is within the range of previous US PSW wages reported ($7.45–17.84 (USD)) (Hewko et al., 2015). For context, the average Canadian hourly wage in 2006 was $19.72, and minimum wage ranging from $6.50–8.25 across provinces. Total earnings from a PSW position averaged $20,784.2 per year (*SD* = 13,202.9); ranging from $17,129 in HC, $21,294 in LTC, to $25,315 in hospital. PSWs typically received higher hourly wages in secondary and tertiary jobs versus their primary PSW job—as indicated by higher hourly wages across all jobs held ($20.70) versus the PSW position alone ($14.00).

**Figure 3.**
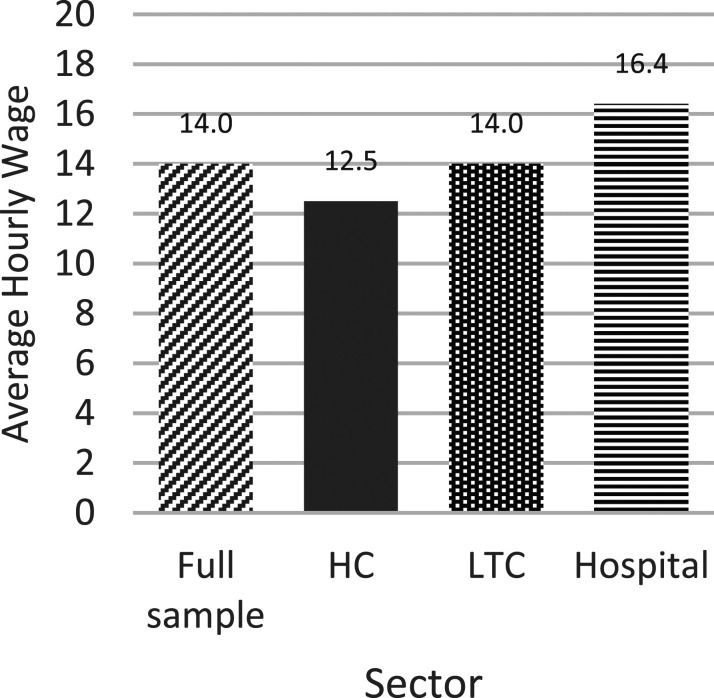
*Average hourly wage adjusted to 2006 for inflation by sector*. Notes: Average hourly wages in Canadian Dollars (CAD) adjusted to 2006 for inflation. Standard deviations for hospital (SD = 4.8), LTC (SD = 4.7), HC (SD = 5.5).

Hourly wage and total earnings significantly differed by sector (*F* (2, 2,179,397) = 210.37, *p* < .01) (*F* (2, 2,179,397) = 144.65, *p* < 0.01). Hospital PSWs had the highest hourly wage (Figure 3) and total earnings and HC PSWs the lowest, coinciding with previous literature from Canada and the US (Crown et al., 1995; Lilly, 2008; Montgomery et al., 2005).

**Figure 4.**
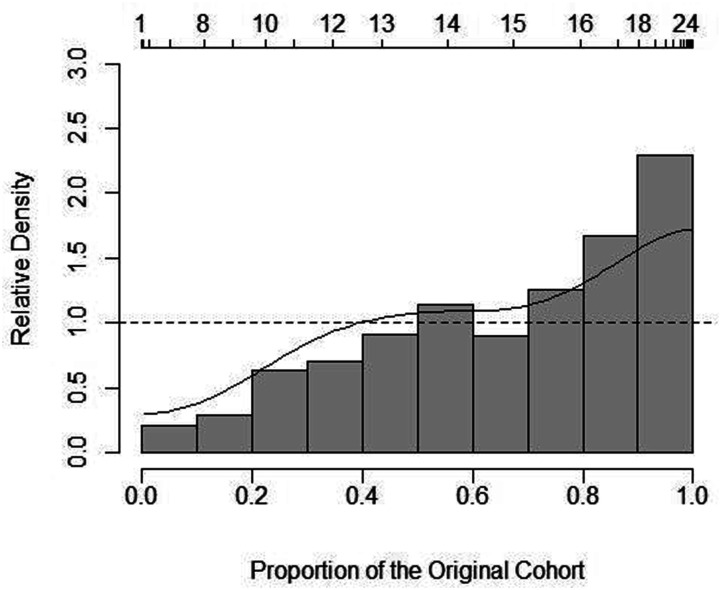
Relative wage distribution adjusted to 2006 for inflation for hospital PSWs versus LTC PSWs. Notes: Relative distribution of PSW hourly wages with LTC PSWs as the reference group and hospital as the comparison group. The upper *x*-axis indicates the true hourly wage values. The lower *x*-axis indicates percentiles of the distribution. The *y*-axis shows the relative density at each point along the distribution. The dashed line at the relative density value 1.0 indicates the point at which any value above indicates greater density by hospital PSWs whereas any value below indicates greater density by LTC PSWs (the reference group).

**Figure 5.**
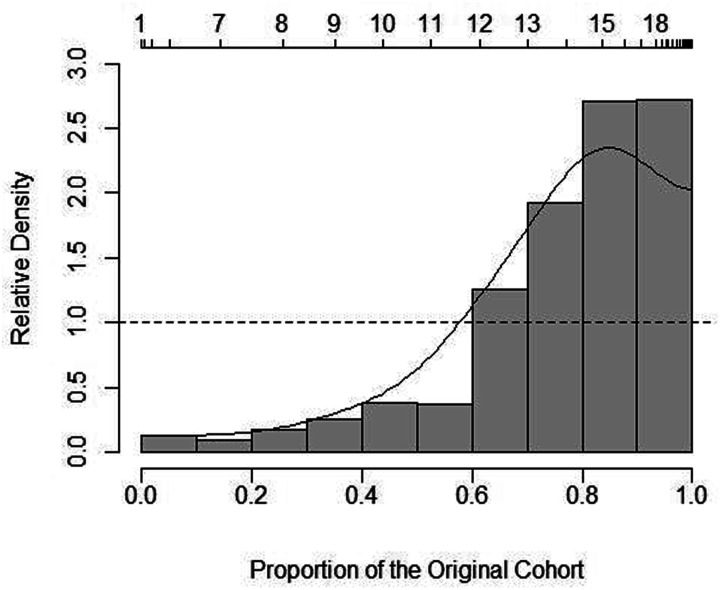
Relative wage distribution adjusted to 2006 for inflation for hospital PSWs versus HC PSWs. Notes: Relative distribution of PSW hourly wages with HC PSWs as the reference group and hospital as the comparison group. The upper *x*-axis indicates the true hourly wage values. The lower *x*-axis indicates percentiles of the distribution. The *y*-axis shows the relative density at each point along the distribution. The dashed line at the relative density value 1.0 indicates the point at which any value above indicates greater density by hospital PSWs whereas any value below indicates greater density by HC PSWs (the reference group).

**Figure 6.**
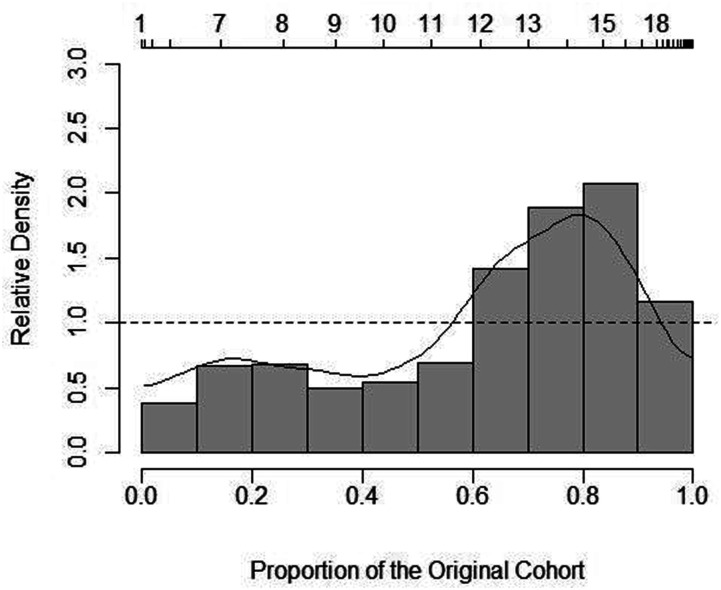
Relative wage distribution adjusted to 2006 for inflation for LTC PSWs versus HC PSWs. Notes: Relative distribution of PSW hourly wages with HC PSWs as the reference group and LTC as the comparison group. The upper *x*-axis indicates the true hourly wage values. The lower *x*-axis indicates percentiles of the distribution. The *y*-axis shows the relative density at each point along the distribution. The dashed line at the relative density value 1.0 indicates the point at which any value above indicates greater density by LTC PSWs whereas any value below indicates greater density by HC PSWs (the reference group).

Relative distribution graphs (Figures 4–6) provide further detail for wage rates by sector. Although hospital PSWs were generally more likely to be paid wages in the higher wage parts of the wage distribution (with greater densities in these areas of the distribution) versus LTC PSWs, LTC PSWs were more likely to receive wages in the mid-range around $14 per hour; this suggests an inconsistency in the otherwise linear relationship of wages between the two sectors. The higher average wage in the hospital sector appears to be driven in part by the payment of much higher-than-average wages to some PSWs in that sector along with a uniformly higher wage being offered compared to PSWs in the other sectors. Between HC and LTC PSW wages, HC PSWs wages tended to be in the lower ranges and LTC the higher. Although at the highest wage range along the distribution ($17 per hour and above), the differences between the relative wage densities of HC versus LTC PSWs was lessened (i.e., closer to 1.0) (Figure 6).

Home and community PSWs consistently had greater wage densities at the lower end of the wage distribution compared to hospital or LTC PSWs. However, note that the densities were larger between HC and hospital (approximately 2.5–3.0 at the highest point (Figure 5)) compared to HC and LTC (approximately 2.0 at the highest point (Figure 6)). Figure 5 provides a visual depiction of the point at which wages shifted from higher likelihood (higher densities) in HC—around $11 per hour—to higher likelihood (higher densities) in hospital PSWs—at approximately $12 per hour and above.

Although the relative distribution graphs echo average wages by sector, the graphs (Figures 4–6) show how the relationships were not always linear and where wages differed along the distribution.

## Discussion

This paper examined multiple variables relevant to PSW labour supply, many of which significantly differed by sector. Overall, hospital PSWs were generally better positioned with more secure jobs in the labour market in terms of attaining FT, full-year, and long-term employment and higher wages, followed by LTC, and lastly HC PSWs.

The relatively casual and transient nature of the HC PSW labour market was reflected in that HC PSWs had relatively lower proportions of permanent positions and lower average job tenure, as well as higher proportions with a secondary job, irregular work schedules, jobless spells, PT, and part-year work versus LTC or hospital based PSWs. It follows that if a policy goal is to attract more PSWs to the HC sector, offering better and comparable employment positions to alternative PSW jobs in other sectors should be considered. From a labour supply perspective, it would be beneficial if the underemployed proportion of part-year, non-permanent positions, or PT PSWs with jobless spells, lower job tenure, and holding secondary jobs—which were especially prevalent in HC—could be retained in the labour market to a more permanent and comprehensive degree. Previous research has found negative implications of many of these job characteristics which were more common for HC PSWs, such as PT positions corresponding to lower wages (Lilly, 2008), and irregular scheduling with worsened health and higher intention to leave the job (Hewko et al., 2015). Future research with more recent data would help to confirm these findings.

Other Ontario-based HC PSW research has reported even lower permanency rates than those found here (74.0% vs. 66.0% previously reported in an Ontario sample (Zeytinoglu et al., 2009)), so that the issue of non-permanent positions may be more pronounced over more recent years and/or within specific provinces. Higher proportions of permanent positions in hospital and LTC sectors may also be indicative of higher skills, qualities, or other characteristics that could be more common in LTC and hospital sectors; however, more research is necessary to capture and examine these characteristics. Moreover, this may suggest differing training requirements for PSWs across care sectors. Indeed, hospital PSWs in this sample had higher levels of education than LTC or HC PSWs.

Some individuals may desire PT or part-year work and therefore choose to work PT or part-year in the HC sector. However, approximately one-fifth of HC PSWs (and LTC PSWs) in this sample wanted to work more weeks. Barriers to achieving FT employment within HC could result from scheduling differences due to increased demand for HC PSWs in the morning and night for many patients—a situation which is less prevalent in institutional settings where 12-hours shifts are more common. Higher proportions of HC PSWs could have been holding a secondary job out of necessity to reach FT hours and corresponding pay.

There may be a higher likelihood of short contract work in the HC sector versus LTC or hospital sector which influenced proportions of permanent status, part-year work, tenure, and jobless spells. Policies that impact the payment structure and extent of contractual funding across sectors would be an area to examine that could help mitigate such sectoral differences. In addition, shorter tenure in HC PSW positions could be influenced by job characteristics—such as lower wages, lower unionization, and different schedules—more common in the HC sector which may lead to higher HC turnover. An area of future research may also be the extent to which the fact that HC services can be contracted to private (for and not for-profit) providers, sometimes in areas that are proximal, which may affect the free movement of PSWs to supply their labour where needed at a given point in time.

Despite non-trivial proportions of unionization amongst PSWs across care sectors, we found that about half of the sample did not have employer-based pension plans (47.5%) and many reported low income (averaging $20,784.2 per year in 2006 (CAD)). As a consequence, some PSWs may continue to work past the age of 65 out of necessity (if physically able) (15% of our sample were over the age of 55) or opt for jobs in another care sector or leave PSW jobs entirely—perhaps even for jobs that offer a lower cash wage if they come with a pension plan. Sectoral discrepancies in pension and income levels indicate that such consequences may be more applicable to HC PSWs than LTC or hospital PSWs.

The average PSW received an income that was close to only the basic needs for a household of three in Canada (the average household size for this sample) in 2006 at $19,673 (Sarlo, 2006); in other words, a good proportion of PSWs may live under the poverty line—a finding which echoes US-based literature (Gleason et al., 2018). Indeed, if an individual left their primary PSW job for their secondary and/or tertiary occupation, they would receive a higher hourly wage ($20.7/hour at primary PSW job vs. $14.0/hour at secondary job). This is highly relevant to PSW supply; it suggests that these workers are open to working in other parts of the economy and thus policy makers and planners need to reckon with the fact that they can expect to compete for PSW workers with other non-health care sectors (e.g., retail). The broad implication is that wages and benefits offered in the health care sector for these workers will have to keep pace with the wages and benefits on offer in the broader economy and that the required levels of compensation are expected to grow given current shortages of workers manifesting in the service industries more broadly.

Similar to prior research, hospital PSWs consistently ranked the highest across multiple income-related variables, while HC PSWs were consistently the lowest. There were likely multiple factors contributing to HC PSWs receiving the lowest wages across sectors. Differences in the socially versus medically aligned historical nature of each sector (Lilly, 2008), variance in PSW education or other skills by sector (where hospital PSWs had higher education in this sample), and/or sectoral processes inherent to each sector may be influencing the contrast in PSW wage by sector. For example, hospital PSWs had higher education and immigrant status in this sample, which may reflect internationally educated nurses working as PSWs in Canadian hospitals who may receive higher wages than less-educated PSWs. It should be noted too that the wage discrepancies reflect long-standing differences in funding levels by sector in Canada. Policies that address pre-existing pay structures across HC, LTC, and hospital may then also improve wages received within HC PSW positions, with a high likelihood of improving HC PSW supply. In addition to receiving lower wages, HC PSWs may also contribute more unpaid hours of work due to lack of appropriate allotted hours per patient, unpaid planning and preparation time, and travel time between clients—the cost of which are often paid out of pocket by the PSW (Home Care Sector Study Corporation, 2003), so that the wage differentials by sector could be even more drastic than shown in the findings as presented here.

### Limitations

Available PSW data is relatively limited; therefore, this data source was one of the few available that captured relevant Canadian PSW characteristics. However, data from more recent years, during or after the COVID-19 pandemic, capturing more specific work variables (e.g., work tasks), PSW-specific work experience, and expanded to capture PSW jobs beyond primary employment would therefore provide further insight.

As with all longitudinal surveys, non-response and attrition could lead to a biased sample. Non-response averaged 24.5% for the entire SLID sample across the time-period (Wei & Wisner, 2010). Consistent with similar surveys, those with low income were more likely to have dropped out of the SLID sample (Boudarbat & Grenon, 2007) and, as HC PSWs are more likely to be in low-income groups than LTC or hospital (Crown et al., 1995; Lilly, 2008; Montgomery et al., 2005), the expected direction of any effects of response rates would be towards expanded sector wage discrepancies.

There is potential geographic variation within each sector that was not reported in this research; wage differences by urban versus rural regions and by province may be expected and further research with larger sample sizes by province could explore this possibility.

## Conclusion

Given the myriad of significant differences found across employer-related characteristics by sector, the PSW labour market may be better described as a set of related, but distinct, sub-markets by sector. It follows that labour supply policies would be most effective if they recognized differences amongst the sectors despite the potential for possible movement of PSWs across sectors, rather than presuming an all-encompassing homogeneous PSW labour market. While the need for improvements across the PSW workforce as a whole is warranted, the findings from this paper point to a relatively higher need for advances in the HC sector to decrease the gap in desirable job and labour characteristics across sectors. The comparatively disadvantaged position that many HC PSWs hold was made especially clear across a multitude of labour characteristics. Increasing the relative attractiveness of the HC sector would be expected to influence retention of HC PSWs, ultimately including the ability to provide care in HC. This is particularly important with the growing demand for HC, as well as other care sectors—not least in response to address increasing care needs for an aging population.
